# Fasting and Exercise in Oncology: Potential Synergism of Combined Interventions

**DOI:** 10.3390/nu13103421

**Published:** 2021-09-28

**Authors:** Rebekah L. Wilson, Dong-Woo Kang, Cami N. Christopher, Tracy E. Crane, Christina M. Dieli-Conwright

**Affiliations:** 1 Division of Population Sciences, Department of Medical Oncology, Dana-Farber Cancer Institute, Boston, MA 02215, USA; Rebekahl_Wilson@dfci.harvard.edu (R.L.W.); Dong-Woo_Kang@dfci.harvard.edu (D.-W.K.); Cameron_Christopher@dfci.harvard.edu (C.N.C.); 2 Department of Medicine, Harvard Medical School, Boston, MA 02215, USA; 3 Department of Epidemiology, School of Public Health, Boston University, Boston, MA 02215, USA; 4 Division of Medical Oncology, Miller School of Medicine, Sylvester Comprehensive Cancer Center, University of Miami, Miami, FL 33146, USA; tecrane@med.miami.edu

**Keywords:** fasting, nutrition, exercise, cancer

## Abstract

Nutrition and exercise interventions are strongly recommended for most cancer patients; however, much debate exists about the best prescription. Combining fasting with exercise is relatively untouched within the oncology setting. Separately, fasting has demonstrated reductions in chemotherapy-related side effects and improved treatment tolerability and effectiveness. Emerging evidence suggests fasting may have a protective effect on healthy cells allowing chemotherapy to exclusively target cancer cells. Exercise is commonly recommended and attenuates treatment- and cancer-related adverse changes to body composition, quality of life, and physical function. Given their independent benefits, in combination, fasting and exercise may induce synergistic effects and further improve cancer-related outcomes. In this narrative review, we provide a critical appraisal of the current evidence of fasting and exercise as independent interventions in the cancer population and discuss the potential benefits and mechanisms of combined fasting and exercise on cardiometabolic, body composition, patient-reported outcomes, and cancer-related outcomes. Our findings suggest that within the non-cancer population combined fasting and exercise is a viable strategy to improve health-related outcomes, however, its safety and efficacy in the oncology setting remain unknown. Therefore, we also provide a discussion on potential safety issues and considerations for future research in the growing cancer population.

## 1. Introduction

The majority of cancer patients at diagnosis, during treatment, and while in remission will experience cancer- and treatment-related physiological and psychological side effects including, but not limited to, undesirable alterations in body composition, increase in cardiometabolic biomarkers, and reductions in quality of life [1]. In many cases, the occurrence of these physiological and psychological outcomes may be more detrimental than the cancer itself and can potentially lead to a poorer prognosis, development of other comorbidities, and pre-mature mortality [2,3,4]. Despite these risks, the benefits of oncology treatments frequently outweigh the risk of side effects [5,6]. While cancer- and treatment-related side effects are likely to occur for majority of patients, the risks of developing severe side effects are often related to patient characteristics such as obesity, functional status, nutritional intake, presence of comorbidities, and genetic predispositions [7,8,9,10,11,12]. With the exception of genetics, these characteristics are largely modifiable via energy balance interventions, which can provide patients with the opportunity to prevent or improve cancer- and treatment-related side effects, resulting in improved overall quality of life.

Energy balance interventions include the manipulation of exercise and dietary habits to alter a person’s energy expenditure and intake, respectively. The adoption of such strategies, albeit tailored to the specific outcome desired (e.g., calorie deficit diet and high volume exercise for weight loss), is growing across various cancer populations and has been shown to improve cancer- and treatment-induced adverse changes in body composition, functional status, inflammatory environments, and prevent obesity-related comorbidities such as cardiovascular disease, type 2 diabetes, and metabolic syndrome [13,14]. Despite many studies highlighting the effectiveness of energy balance interventions for cancer patients, there is still much debate about the most appropriate prescriptions. Combined fasting and exercise is one such prescription that is growing in interest within the oncology field based on their respective independent benefits.

Fasting is the purposeful avoidance of food, and in some cases drink, for a specific amount of time. Fasting is an ancient practice and has been used for medical purposes since the fifth century B.C. with Hippocrates recommending abstinence from food or drink for patients presenting with specific symptoms of illness [15]. Today, fasting is practiced for a variety of reasons including religious, health, and ritualistic purposes (Table 1). Within oncology, fasting has been evaluated as a potential intervention to alleviate treatment-related toxicities and symptoms, and to potentially impact body composition and cardiometabolic outcomes in people with cancer [16,17]. Despite a limited number of trials with the majority including small sample sizes, studies have found fasting to be a safe intervention while receiving treatment for cancer [18,19,20,21]. Long-term fasting (e.g., several weeks/months of reduced energy intake with fasting periods >72 h), may not be feasible in oncology care due to its potential to increase risk of undesirable weight loss in cancer patients [22]. However, shorter-term fasting (e.g., interventions completed over several weeks/months utilizing fasting periods of 12 to 72 h with ad libitum feeding during fed hours) may be feasible for cancer patients. It is important to note that fasting-mimicking diets have been studied as a mechanism to improve risk factors associated with cancer-related outcomes [23,24], however, this type of fasting differs from intermittent fasting in that they do not have specified windows of eating, but rather promote a low-calorie diet with a specific meal plan, as such this type of diet is not discussed in the current review. Exercise has also been deemed a safe and feasible intervention within various oncology settings with the majority of current evidence in breast, prostate, lung, and colorectal patients [14]. While not yet a consistent strategy utilized within oncology standard of care, there is strong evidence indicating exercise improves health-related quality of life, various psychosocial outcomes such as anxiety and depression, cancer-related fatigue, and cardiorespiratory fitness and muscular strength among various cancer patients on treatment and in remission [25,26]. Furthermore, although preliminary, several studies have reported that exercise may play a role in improving treatment tolerance and efficacy (e.g., relative dose intensity and treatment delivery, tumor size, and long-term survival) [27,28,29,30,31].

As a combined entity, fasting and exercise have been shown to induce synergistic effects on improved metabolic outcomes such as body composition, cholesterol, and insulin sensitivity in non-cancer populations [34]; although much of the research examining the interaction of fasting and exercise has been carried out in sports performance and acute settings [35]. However, there is a growing interest for how combined fasting and exercise interventions may be optimized for health and therapeutic benefits in oncology settings, based on the current evidence in the non-cancer population and their independent benefits established within the cancer population [14,35,36,37]. In this review, we summarize the current evidence on the independent effects of fasting and exercise in cancer settings and discuss the potential impacts and mechanisms of combined fasting and exercise interventions on cardiometabolic, body composition, patient-reported outcomes, and cancer-related outcomes. We also discuss the potential safety issues of combined exercise and fasting in cancer patients and suggest considerations for future research in this setting.

## 2. What Metabolic Changes Occur during Fasting and Exercise?

The combination of fasting and exercise can drastically change how our bodies utilize and synthesize fuel sources. It is important to understand the physiological changes that occur during this state before identifying the potential beneficial outcomes that a combined fasting and exercise strategy may induce among cancer patients.

The act of consuming food provides our bodies with nutrients that are broken down and utilized as fuel in order to survive. However, when food is not supplied, the body relies on processes of biosynthesis as well as stored glycogen, fats, and proteins as metabolic fuel substrates [38]. Therefore, the metabolic substrates, and their catabolic or anabolic pathways, differ in a fed state from a fasted state and is likely one of the key mechanisms responsible for many of the changes observed when undertaking fasting compared to a fed intervention [39,40]. In a fed state, the body predominantly utilizes glucose from the recently consumed meal as the primary source of fuel via glycolysis, oxidative phosphorylation, and carbohydrate oxidation [38,41,42]. In a fasted state, glucose and glycogen stores are depleted, so fats become the primary source of fuel via lipolysis and fat oxidation [38,42,43]. Gluconeogenesis and ketogenesis are also increased in a fasted state to ensure the homeostasis of organs that only use glucose or ketones as fuel [38].

The preferential fuel source of the body is further altered when exercise is included and is dictated by exercise completed in a fed or fasted state, the type of meal consumed before exercise, and the intensity and duration of exercise [44,45]. For example, when exercising in a fed state, glucose is the predominant source of fuel; however, when compared to consuming a meal with a high glycemic index (GI), a low GI meal is associated with lower rates of glycolysis [44]. Fuel for exercise in a fasted state comes from increased fat oxidation, particularly the breakdown of intramyocellular triacylglycerol (IMTG) [43]. Regardless of a fed or fasted state, the metabolic substrate utilization is similar at intensities >70% of VO_2max_ or durations of continuous exercise >2 h [45]. These acute differences in metabolic substrate utilization highlight that timing of a fasting period and exercise bout, when undertaking a combined fasting and exercise intervention, may be of critical importance as it is unclear how timing will impact the long-term benefits of a combined intervention [36].

## 3. Effect of Fasting, Exercise, and Combined Fasting and Exercise

**Figure 1:**
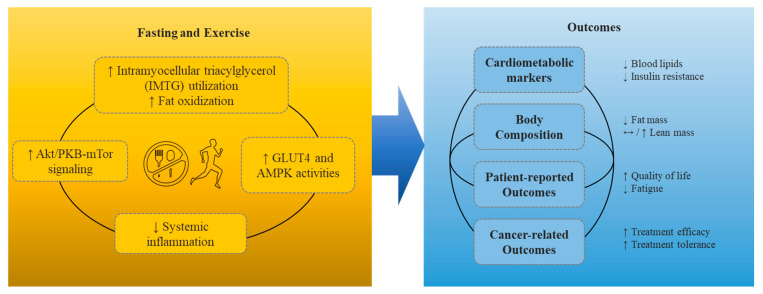
Potential mechanisms and impacts of combined fasting and exercise in cancer patients. Up arrow refers to an increase in the component, down arrow refers to a decrease, and the side ways arros refer to no change.

The potential mechanisms and impacts of combined fasting and exercise in cancer patients are illustrated in Figure 1. Briefly, exercise during a fasted state in cancer patients can maximize glucose regulation, lipid oxidization, and systemic inflammation to improve each of the suggested outcomes including cardiometabolic markers, body composition, patient-reported outcomes, and cancer-related outcomes. We discuss below the independent mechanisms and effects of fasting and exercise and then the effects of potential benefits of combined fasting and exercise by each outcome.

### 3.1. Cardiometabolic Biomarkers

With advances in diagnosis programs, treatments, and general awareness of cancers and their signs and symptoms, the 5-year survival rates of many cancer types have improved over recent decades [46]. However, systemic cancer treatments such as chemotherapy and hormonal therapy, may induce cardiometabolic dysregulation (e.g., insulin resistance, dyslipidemia), which can also lead to the development of other comorbidities, poorer quality of life, and sometimes pre-mature mortality [4]. Therefore, it is important to identify interventions that can improve cardiometabolic biomarkers such as insulin-related markers and lipid profiles.

#### 3.1.1. Fasting

As previously discussed, when in the fasted state the body transitions to utilizing glycogen stores for energy in an effort to maintain glucose homeostasis. Insulin-like growth factor-1 (IGF-1) is a primary mediator of growth hormone and has significant metabolic effects. Obesity has been attributed to 15–20% of cancer-related deaths where obese individuals often present with higher levels of IGF-1 which has been identified as a potential mechanism associating obesity with increased cancer risk and disease progression [47,48,49]. Several studies utilizing short-term fasting have demonstrated a reduction in IGF-1 with some also observing a reduction in insulin [50]. In women with breast cancer, data analysis from the Women’s Healthy Eating and Living Study showed that each 2 h increase in overnight fasting was associated with a significant reduction in hemoglobin A1C (HbA1c) (β = −0.37; 95% CI, −0.72 to −0.01) [51]. A growing body of literature suggests short-term fasting may play a role across the cancer continuum to improve outcomes, such as treatment toxicity and efficacy, through normalization of metabolic markers, with several clinical trials of short-term fasting underway in cancer patients [21]. Importantly, obesity and high levels of insulin and IGF-1, in addition to a diagnosis of diabetes mellitus are associated with worse survival in cancer [52,53,54,55]. While the current evidence is mostly focused on insulin and glucose pathways, the effects of fasting on lipid profiles are underexplored in cancer settings, which warrants further investigations.

#### 3.1.2. Exercise

Cardiometabolic changes are widely studied within the oncology field as many systemic cancer treatments result in side effects that alter metabolic homeostasis (e.g., androgen deprivation therapy causing insulin resistance) [56,57]. Such metabolic dysregulations can have a devastating effect and lead to an increased risk of cancer progression, most commonly documented for breast, colorectal, prostate, and endometrial cancers, and development of other comorbidities, in direct and indirect mechanisms including obesity [58,59]. Aerobic exercise increases the rate of glucose uptake into the contracting skeletal muscles, up to 2–3 fold, primarily through the regulation of GLUT4 glucose transporters, which improves insulin sensitivity and lowers circulating insulin levels [60]. Resistance training that induces muscle hypertrophy and qualitative adaptation can also improve insulin resistance by enhancing the expression of glucose transporters and mitochondrial oxidative capacity [61]. More evidence, therefore, supports the synergistic effects of aerobic and resistance training on insulin sensitivity and glucose metabolism [62]. For blood lipid levels, oxidation of triglycerides and fatty acids progressively increases during aerobic exercise to generate energy sources, especially at a lower intensity (~45% of VO_2max_), however, this process heavily depends on the individuals’ fitness and the rates of other substrate oxidation at different exercise intensities [63]. Resistance exercise may elicit lower energy expenditure during a single bout of exercise than aerobic exercise [64], however, the training effects of resistance exercise, including maintenance or improvement of muscle mass and density, higher resting metabolic rate, and increased fat metabolism, also contribute to the reduction in circulating lipid levels (e.g., reduced total cholesterol, low-density lipoprotein (LDL), and triglycerides, and increased high-density lipoprotein (HDL)), which further establish the additive benefits of combined aerobic and resistance exercise [65,66].

#### 3.1.3. Combined Fasting and Exercise

There is no current evidence describing the effect combined fasting and exercise interventions have on lipid levels in the cancer population. Therefore, we draw on non-cancer examples to propose the potential benefits a combined intervention may have on cardiometabolic outcomes of cancer patients. Exercising in the fasted state, compared to a fed state, appears to be more effective at manipulating lipid levels given the increase in lipoprotein lipase activity in the non-cancer population [67]. Bhutani et al. [68] assessed the effect of a 12-week combined fasting and exercise intervention on lipid levels of non-cancer obese participants utilizing aerobic exercise and alternate day fasting where 25% of their daily energy intake was consumed on the “fast” days between 12–2 p.m. and ate ad libitum on the “fed” days. The authors reported no change in total cholesterol or triglyceride concentrations for any group; however, the combined fasting and exercise group significantly reduced LDL (−12 ± 5%) and increased HDL (18 ± 9%) concentrations, compared to no change in the fasting-only, exercise-only, and control groups. The combined fasting and exercise group also demonstrated favorable changes in the proportion of small LDL and HDL particles, further emphasizing the cardio-protective effects of this type of intervention, compared to either fasting or exercise alone. In contrast, Cho et al. [69] did not find any changes in total cholesterol, LDL, and HDL between groups, although they did employ a shorter intervention of 8 weeks utilizing combined aerobic and resistance exercise but had a similar alternate day fasting regime as Bhutani et al. [68]. However, Cho et al. [69] did report a significant difference in triglyceride concentrations where combined fasting and exercise-induced a decrease, compared to an increase for the control group. Both studies significantly decreased fat mass, so the change in lipid levels cannot be attributed to the intervention itself, but is potentially dependent on fat mass change. This has been suggested to be the case among cancer patients where men with prostate cancer on androgen deprivation therapy (ADT) undertaking an exercise-only study demonstrated an improvement in triglycerides, but was dependent on a loss in fat mass [70]. Further research is required into the best prescription to manipulate lipid levels in cancer patients, particularly as it relates to the timing of a meal in a fasting and exercise intervention, and if a loss in fat mass is required to alter the lipid profile.

Evidence suggests that an increase in total fat mass, with a particular impact of high concentrations of IMTG, is a significant contributor to the development of insulin resistance in both cancer and non-cancer populations and could be a key target area when trying to improve metabolic outcomes [36,71,72,73]. The timing of an exercise bout in relation to the fed-period may be of high importance when trying to optimize the impact of a combined fasting and exercise intervention on fat mass and IMTG to improve insulin resistance and glycemic control given the changes in substrate utilization experienced in the varying fasted, fed, and exercising states [67]. Combined fasting and exercise can significantly reduce total fat mass, as described later in the body composition section [68,69]. Similarly, an acute bout of exercise in a fasted state, compared to a fed state, can induce a ~60% depletion in IMTG in type I muscle fibers, as it is a readily available source of fats for fuel [43,74]. Consequently, these superior changes in fat mass and IMTG may contribute to desired improvements in insulin resistance. For example, within the non-cancer population, Cho et al. [69] reported no between-group differences in HOMA-IR, a measure of insulin resistance, across four assessed groups (combined fasting and exercise, fasting-only, exercise-only, and control), although there was a trend for a between-group difference at baseline (*p* = 0.063) where those in the combined intervention group had a mean score of 2.23 ± 0.72, and the control group 0.97 ± 0.45. However, over the 8-week intervention, the combined fasting and exercise group had a non-significant mean decline of −1.12 ± 0.68, whereas the control group had a significant 1.10 ± 0.97 increase. While there are no standardized cut-off points for insulin resistance as defined by HOMA-IR, a score <1.5 has consistently been identified as insulin sensitive with superior metabolic outcomes [75,76,77,78]. Therefore, the observed change, while not statistically significant, may be of clinical relevance and needs to be further evaluated, particularly within the context of fat mass loss given the combined fasting and exercise group lost significantly more fat mass than the control (−3.2 ± 0.5 versus −0.3 ± 0.8 kg), although IMTG was not examined. Furthermore, it is unclear if the exercise bout in the combined fasting and exercise group was completed on fasting days only, feeding days only, or a combination. Understanding the timing of fasting and exercise as well as meal consumption prior to exercise may provide further insights into why we see participants who respond and do not respond to lifestyle interventions and is an important factor to consider during a combined fasting and exercise intervention.

Another potential mechanism for combined fasting and exercise to improve insulin resistance and glycemic control is through the increase in GLUT4 protein and AMPK activity. Van Proeyen et al. [79] recruited young healthy male participants who all undertook a 6-week hypercaloric high-fat diet and were randomized to either high-fat diet control, exercise in a fasted state, or exercise in a carbohydrate-fed state where both exercise groups completed 4 aerobic exercise sessions per week at ~70–80% heart rate maximum. The authors reported that exercise in a fasted state alleviated the negative effects of a high-fat diet on glucose tolerance and insulin sensitivity, and was attributed to the increase in GLUT4 protein and AMPK activity in the fasted exercise group, compared to no change in the carbohydrate-fed exercise, and high-fat diet control groups. However, it is unclear if similar long-term results would be observed if the carbohydrate-fed exercise group were to also undertake a fasting component, yet, still eat prior to exercise. Nevertheless, this study highlights that in the absence of healthy dietary advice and an iso- or hypocaloric state, undertaking exercise after an overnight fast has a positive effect on insulin sensitivity and glycemic control. This type of intervention is worth further exploring for cancer patients who may be eating poorly and do not want to change their dietary intake given they are already undergoing a substantial number of changes, as such a fasting component with no dietary advice may result in increased compliance to the intervention. However, the benefit of improving dietary intake should not be completely dismissed.

### 3.2. Body Composition

An increase in fat mass and decline in lean mass loss often occurs in cancer patients as a result of hospitalization and extended bedrest, increased stress-related eating, decreased physical activity, or as a result of treatment [80,81]. Within the cancer population, the quantity and distribution of fat mass and lean mass are influential in the effectiveness of treatment, development and severity of cancer- and treatment-related side effects, and progression of cancer [80,82,83,84]. Changes in fat mass and lean mass can also play critical roles in the development of cardiometabolic outcomes, previously described, and contribute to the development of comorbidities and further reducing the health and well-being of a cancer patient [2,3,4]. Therefore, implementing lifestyle-based intervention strategies, such as fasting and exercise, to improve body composition, or prevent cancer- or treatment-induced worsening of body composition, is critical to a cancer patient’s care. Here, we discuss the impact fasting, exercise, and combined fasting and exercise interventions have on fat mass and lean mass among cancer patients and the potential mechanisms involved.

#### 3.2.1. Fasting

Body composition changes as they relate to fasting appear to be associated with the length of time fasting occurs. In a pilot crossover study among cancer patients undergoing chemotherapy, comparing cycles of short-term fasting to normocaloric diet, a significant loss in mean fat mass (measured by bioelectrical impedance) (−0.63 ± 0.23; 95% CI −1.09–(−0.17); *p* = 0.008) was observed which lead to a significant weight loss during moderate short-term fasting (−0.84 ± 0.26; 95% CI −1.35–(−0.33); *p* = 0.002) [50]. Aside from fat mass, body composition remained stable. Mean body weight and mean fat mass were 71.4 ± 12.3 kg and 23.0 ± 8.8 kg at the beginning and 69.8 ± 11.6 kg and 21.4 ± 8.4 kg at the end of the intervention, respectively [50]. Other studies corroborate these findings for lack of weight change using short-term fasting [16]. In these studies, after the fasting days, an increase in weight was commonly resulting in achievement of baseline weight [16,50,85]. These early studies suggest interventions that include short-term fasting carry a low risk of negatively impacting body composition and therefore likely the better option for cancer patients, particularly those at risk of weight loss leading to a poor prognosis (e.g., lung cancer).

#### 3.2.2. Exercise

The changes in body composition followed by exercise training have been widely investigated in cancer settings. Particularly, most exercise oncology research with body composition outcomes has focused on lean mass in prostate cancer patients receiving ADT [86], where patients often experience significant declines in lean mass [87,88]. A recent meta-analysis of 21 clinical trials in prostate cancer patients reported that exercise, primarily resistance exercise, significantly reduced fat mass by 0.6 kg and increased lean mass by 0.5 kg after a mean intervention period of 20 weeks [89]. Resistance exercise is effective in improving, or at least maintaining, lean mass by counteracting impaired anabolic signal pathways and inhibiting the cellular atrophy mechanism during ADT [90]. Fat mass has been more commonly investigated in patients with breast or colon cancer given the strong links between adipocytokines/obesity-related markers and these cancers [91]. Although several studies have demonstrated the significant loss of fat mass after exercise [92,93,94,95,96], this is commonly a result of the control group continuing to increase fat mass as opposed to the exercise intervention inducing a significant fat mass decline. Additionally, fat mass is more commonly a primary outcome of interest in combined physical activity (e.g., meeting physical activity guidelines) and dietary studies [97,98]. Overall, there is generally a lack of evidence on body composition outcomes other than ADT settings, and the findings are not consistent and heavily depend on the modes and intensities of exercise [86,99].

#### 3.2.3. Combined Fasting and Exercise

Given the strong connection between obesity, or excess fat mass, and cancer development and progression, weight loss, or more importantly fat mass loss, is often a key consideration as part of a cancer patient’s care [100]. By combining fasting and exercise, there is some evidence that such an intervention will have a synergistic effect on fat mass loss due to increased fat oxidation and energy expenditure over intake [68,69]. Within the non-cancer obese population, two randomized control studies have been conducted where body composition changes were compared between four groups: combined fasting and exercise, fasting-only, exercise-only, and a control group [68,69]. Cho et al. [69] reported fat mass to significantly decrease in both the combined intervention (−3.2 ± 0.5 kg) and fasting-only (−3.2 ± 0.6 kg) groups compared to control (−0.3 ± 0.8 kg). The exercise group also significantly decreased fat mass (−1.7 ± 0.5 kg) compared to baseline, but not the control group. While Bhutani et al. [68] also reported both the combined intervention and fasting-only groups to reduce fat mass, the combined intervention group had a superior loss (−5.0 ± 1.0 kg versus 2.0 ± 1.0 kg). While the studies utilized similar alternate day fasting regimens, they differed in exercise modes (combined aerobic and resistance versus aerobic-only) and lengths of intervention (8 versus 12 weeks), which likely contributed to the variation in fat mass results. These studies indicate the potential feasibility of a combined fasting and exercise intervention to improve fat mass in obese individuals, but how this translates to the obese cancer population is unknown. It must be highlighted that combined fasting and exercise can induce fat mass loss, independent of total weight loss, by prescribing an energy balance or surplus during feeding hours [79,101]. This is important for the cancer population as weight loss can sometimes be a red flag for poor prognosis (e.g., cachexia), or is not recommended during treatment such as radiation therapy as it could result in day-to-day movement of organs, therefore, decreasing radiation accuracy if image-guided radiation is not used [102,103]. Further research is required into the best prescription of combined fasting and exercise interventions for cancer patients and its effect on fat mass changes both dependent and independent of total weight loss.

The pathways involved in lean mass changes (e.g., Akt/PKB-mTor signaling) are down-regulated in fasting-only interventions leading to an increase in muscle protein breakdown; however, with the addition of exercise, Akt/PKB-mTor signaling is reactivated, leading to lean mass maintenance, although this has only been shown in murine models [34]. The ability to significantly increase lean mass while undertaking a combined fasting and exercise intervention is unclear. Resistance exercise and protein supplementation are known strategies to induce lean mass hypertrophy [104,105]. However, while in an energy deficit state, anabolic suppression (i.e., a blunted training response) during resistance training has been previously demonstrated even in the presence of protein supplementation and adequate daily protein intake of 1.2 g·kg^−1^ body weight and may explain the lack of lean mass hypertrophy reported in combined fasting and exercise interventions [106,107]. In the same way, fasting and exercise interventions may be able to reduce fat mass independent of weight loss by manipulating energy intake during fed hours, the same concept may apply to achieving an increase in lean mass. Tinsley et al. [108] examined non-cancer resistance-trained females and compared three groups over 8 weeks: combined fasting and resistance exercise with a calcium β-hydroxy β-methylbutyrate supplement, combined fasting and resistance exercise with a placebo, where both groups undertook time-restricted fasting regimens, and a non-fasting control diet with a placebo. All groups also received daily protein supplementation. The study demonstrated a significant increase in lean mass (1.0–1.4 kg) over the 8-week period for all groups compared to baseline with no between-group differences. All groups significantly increased their total energy intake during the intervention, which may have contributed to the significant increase in lean mass observed in contrast to other combined fasting and exercise studies that had an energy balance or deficit [40,68,69]. Moreover, this study was conducted in young, trained female participants and its applicability to the male, un-trained, and cancer populations is limited. Further research is required to examine the best prescription of exercise mode, nutrient supplementation, and total energy intake to induce a significant increase in lean mass while undergoing a combined fasting and exercise intervention.

### 3.3. Patient-Reported Outcomes

Pain, fatigue, anxiety, depression, and sleep disturbances are among the most commonly identified detrimental patient-reported outcomes of cancer and cancer-related treatment [109,110,111]. Here, we discuss the impact of fasting, exercise, and combined fasting and exercise-based interventions have on these outcomes in cancer patients and the potential mechanisms involved.

#### 3.3.1. Fasting

Commonly experienced symptoms as a result of cancer and its treatment including fatigue, gastrointestinal disturbances, and pain have all been preliminarily examined as potential patient-reported outcomes that may be improved as a result of short-term fasting. A case series of 10 patients with various types of cancer demonstrated that fasting in combination with chemotherapy is feasible and eluded to the potential for fasting to reduce fatigue, weakness, and gastrointestinal side effects [20]. In a pilot study among breast and ovarian cancer patients undergoing chemotherapy, women randomized to either undergo short-term fasting in the first half of their chemotherapy cycle followed by their usual diet or vice versa with short-term fasting followed in the second half of the chemotherapy cycles. For both groups in the fasted state, quality of life and fatigue scores both improved [16]. A pilot study by Zorn et al. [50] found a modified short-term fast during chemotherapy reduced stomatitis, headaches, weakness, and overall total toxicities score. Although there have been a limited number of studies, initial findings of the impact of fasting on patient-reported outcomes in cancer are promising.

#### 3.3.2. Exercise

There is strong evidence on the benefits of exercise on numerous patient-reported outcomes during and after cancer treatment [25], such as health-related quality of life [112,113], cancer-related fatigue [114,115], and anxiety and depression [116]. The mechanisms of the positive impacts of exercise on psychosocial distress may include direct psychological interpositions, such as providing a constructive distraction and reducing time on rumination, directing energy positively, and improving the feeling of control over cancer [117], as well as biological pathways, such as releasing β-endorphins and circulating levels of neurotrophic factors (BDNF) [118]. For cancer-related fatigue, engaging in exercise, although counterintuitive, plays a significant role in reducing acute and chronic fatigue, which is superior compared to paratheatrical agents or psychological interventions [115]. Potential mechanisms include reducing elevated pro-inflammatory cytokines, normalizing circadian rhythm dysregulation, and improving impaired muscle oxidative capacity [119].

#### 3.3.3. Combined Fasting and Exercise

Independently, both fasting and exercise are associated with improved patient-reported outcomes (e.g., quality of life, fatigue, depression), in both cancer and non-cancer populations [16,120]. However, the effect of a combined fasting and exercise intervention on patient-reported outcomes is not well described. The study by Albrecht et al. [37], described further in the cancer-related outcomes section, is the only study that describes the effect of combined fasting and exercise on patient-reported outcomes among cancer patients. The ovarian cancer patient that was examined in this case study reported an improvement in feelings of anxiety, perceived stress, and emotional functioning. While this case study highlights the potential for a combined fasting and exercise intervention to improve patient-reported outcomes, it cannot be dismissed that an improvement was observed due to the feeling of hope that may have come from entering a study with the intention of improving disease outcomes.

### 3.4. Cancer-Related Outcomes

Lifestyles that contain an increased amount of physical activity and the consumption of a healthy diet are well-established modifiable factors that decrease a person’s risk of cancer development [121]. Given this relationship, research has increased in examining the role of exercise and nutrition after a cancer diagnosis in the progression of cancer and the effectiveness of cancer-related treatment. Termed the Warburg Effect, cancer cells rely on aerobic glycolysis deriving most of their energy from glucose converted to lactate for energy followed by lactate fermentation, even when oxygen is available [122]. As such, a shift in energy metabolism from glycolytic metabolism to oxidative phosphorylation, which occurs in a fasted state, may be a means by which cancer growth rate is altered [123]. Therefore, combined fasting and exercise has the potential to provide this needed change in metabolism to combat cancer [21,124]. Here, we discuss the impact fasting, exercise, and combined fasting and exercise-based interventions have on cancer progression and recurrence, and treatment tolerance and effectiveness and the potential mechanisms involved.

#### 3.4.1. Fasting

Broadly in humans, studies of long-term calorie restriction, including or excluding long-term fasting periods, have demonstrated a reduction in metabolic and hormonal factors associated with cancer risk [125,126,127]. However, long-term fasting (e.g., >72 h) is not practical in the oncology care space as it may lead to unacceptable weight loss in cancer patients [22]. Short-term fasting (e.g., 12–72 h) may be feasible for cancer patients. In mice, shorter periods of fasting have been shown to slow cancer growth as effectively as long-term fasting without compromising body weight [128,129,130] with the effects of the short-term fasting improving differential stress response between healthy somatic cells and cancer cells [19,128,129,131,132]. The mechanism by which this is occurring is through a protective response in healthy cells wherein nutrient deprivation (fasting) shuts down pathways promoting growth in order to provide energy in maintenance and repair pathways that contribute to resistance to chemotherapy, a phenomenon knowing as ‘differential stress resistance’ [16,133,134]. Alternatively, due to mutations in oncogenes, tumor cells are unable to activate this protective response because of uncontrolled activation of growth pathways. In order for tumors to maintain their high rate of growth, an abundance of nutrients are required and thus short-term fasting leads to increased sensitivity of tumor cells to chemotherapy [128,129,130]. This increased sensitivity is hypothesized to be a promising strategy to enhance the efficacy and tolerability of chemotherapy [19]. For example, in another study examining the feasibility of dose escalation fasting (24, 48, and 72 h) over the course of a chemotherapy cycle, patients who fasted for ≥48 h had a trend towards reduced neutropenia compared to patients who only fasted for 24 h periods with the 48 h fasting group also reducing leukocyte damage [18]. In a pilot study of short term fasting in HER-2 negative breast cancer patients, those who were randomized to the short term fasting intervention, compared to unfasted women, experienced reduced hematological toxicities 7 days post-chemotherapy administration (*p* = 0.007, 95% CI 0.106–0.638 and *p* = 0.007, 95% CI 38.7–104 for erythrocyte and thrombocyte counts, respectively) [19]. Patients undertaking short-term fasting, compared to non-fasted patients, have also been shown to have fewer postponements of chemotherapy [50]. Finally, in a secondary analysis of women with breast cancer participating in the Women’s Healthy Eating and Living Study women who fasted <13 h/night had a 36% increased risk of recurrence (HR, 1.36; 95% CI 1.05–1.76) compared to those who fasted ≥13 h per night [51].

As use of immunotherapy increases in oncology, fasting demonstrates some promise in preclinical studies as a potential modality to bolster antitumor immunity. Prolonged overnight fasting was found to reduce IGF-1 levels and protein kinase A activity in a variety of cell populations in mice leading to signal transduction changes in long-term hematopoietic stem cells [135]. Further, multiple cycles of fasting lessened immunosuppression and chemotherapy-induced mortality. In both in vivo and in vitro studies in mice with colorectal cancer, alternate day fasting for two weeks inhibited tumor growth without causing a reduction in body weight, suppressed M2 polarization of tumor-associated macrophages inhibiting tumor growth through decreased levels of adenosine, and increased autophagy of tumor cells [135,136]. Further research is required into the differing effects that such types of fasting may have on cancer-related outcomes.

#### 3.4.2. Exercise

A body of preclinical evidence has demonstrated the direct impacts of exercise in suppressing tumor progression and metastasis [137,138], yet, evidence within the clinical setting is lacking. The underlying mechanisms are still unclear, however, several plausible mechanisms include the acute increase in the concentrations of immune cells (e.g., natural killer cells, monocytes, and neutrophils), the muscle-to-cancer crosstalk through muscle contraction-derived cytokines (e.g., interleukin-6 and SPARC), and the downregulation of tumorigenesis pathway through catecholamine (e.g., epinephrine). These mechanisms also interdependently suppress tumor growth by enhancing mobilization and redistribution of cytotoxic immune cells into the tumor cells [139,140]. Another mechanism that has been identified in which exercise can improve cancer-related outcomes is through increased tumor vascular permeability and angiogenesis. Recent preclinical studies showed that repeated bouts of aerobic exercise enhanced treatment efficacy and thereby suppressed clinical tumor progression by improving tumor vascular permeability and angiogenesis, which caused oxygen delivery and drug penetration into tumor cells [141,142]. This mechanism is plausible as hypoxic status is one of the key characteristics of tumor microenvironment (TME), which increases treatment resistance to the tumors and can be reversed by the improvements of vascular functions during aerobic exercise. Lastly, emerging evidence has demonstrated that maintaining or improving lean mass during chemotherapy may improve chemotherapy tolerance and completion in cancer patients [27,143]. Systemic cancer drugs are primarily distributed and metabolized (i.e., pharmacokinetics) by blood flow and perfusion in lean tissues, however, treatment dosages are typically determined by estimated total body surface area (BSA) without considering body composition [144,145]. Cancer patients with identical BSA may present substantial differences in body composition, which is associated with chemotherapy toxicity and efficacy [146]. Therefore, resistance training to improve body composition (i.e., increased lean mass and decreased fat mass) as well as potentially muscle quality (i.e., reduced IMTG content) [88] poses a great potential to enhance treatment outcomes. Nevertheless, only preliminary evidence exists and very little is known about how exercise may mediate the response to cancer therapy in patients, where further preclinical and clinical exercise research is warranted.

#### 3.4.3. Combined Fasting and Exercise

Despite the independent impacts of fasting and exercise, the effect of a combined fasting and exercise intervention on cancer-related outcomes is largely unknown. To our knowledge, only one study has been conducted in the cancer population that utilized a combined fasting and exercise intervention [37]. This proof of concept case study, which examined a woman with recurrent stage III ovarian cancer in a watch and reevaluate phase of treatment, evaluated the effect of the intervention on ovarian tumor growth as well as health-related quality of life and psychological symptoms. The intervention involved an 18 h fast, low-fat meal, flaxseed oil and caffeine supplements, and 90 min of treadmill walking repeated daily across a 3-day period where the patient was housed in a research facility and completed once a month for 3 months. This intervention was selected to slow cancer progression based on the proposed mechanisms where it would create the best environment to induce the optimal free fatty acid (FFA) level of 1 to 2 nM maintained over an extended period of time, and that unsaturated fats (flaxseed oil) has a cytotoxic effect having been demonstrated in preclinical studies [147,148]. On the days where emesis did not occur, four out of seven of the study days, FFA concentrations reached this desired level for ≥4 h. However, CA125, a marker used to monitor ovarian cancer progression, continued to increase over the course of the study period, although a computed tomography scan indicated no sign of cancer progression. Given the study design, conclusions about the effect a combined fasting and exercise intervention has on tumor outcomes is limited. The role FFAs play in cancer prognosis is complex and the mechanisms are not fully understood [149]. Further research is required into the previously identified independent mechanisms of fasting and exercise, and how combining these interventions may have a superior, synergistic effect, in altering TME and treatment tolerance and efficacy.

## 4. Safety with Intervention Implementation

Though fasting and exercise have independently been shown to have low adverse events and are generally safe in cancer populations, intervention safety should be addressed for future research and implementation. When various periods of fasting were utilized prior to and up to 24 h post-chemotherapy, patients commonly reported negative symptoms including headaches, nausea, dizziness, and fatigue, though these were not severe enough to be considered an adverse event [18]. Additionally, when intermittent fasting is not managed, it can cause malnutrition, eating disorders, and severe damage to organs [150]. It is unclear how exercise in combination with fasting may escalate these negative outcomes among cancer patients, particularly during treatment. Furthermore, the combination of exercise and fasting may be detrimental in maintaining body composition for patients who already have a low BMI or cachexia. The risks of being underweight, compounded with the possible combined impact of fasting and exercise on weight loss and fat mass loss, may further impair treatment efficacy and result in a poorer prognosis [102,103]. Given these safety concerns, it is crucial that future studies are thoroughly designed to mitigate these risks and to promote the prospective desired health benefits of fasting and exercise among cancer patients.

## 5. Future Research and Key Considerations

The sparsity of research with multimodal fasting and exercise interventions among cancer survivors lends to a plethora of future investigations to improve research in this area.

### 5.1. Timing of Intervention Delivery

While studies have shown that exercise after an overnight fast has beneficial effects on insulin sensitivity and glycemic control, it is not clear how the timing of treatment may interact [36,79]. Long-term impacts of the timing of this relationship of fasting and exercise are not well established with respect to treatment. It is also not clear at what point in the cancer diagnosis trajectory that this combined intervention may be most beneficial. Perhaps an opportune time to intervene includes an emphasis on the pre-surgical window. For example, there may be a benefit to a staggered approach of intermittent fasting prior to surgery followed by exercise or there may be synergy between the two modalities that would prove advantageous to improving the TME between diagnosis and surgery.

### 5.2. Alternative Intervention Modalities

Consideration of the individual components of a lifestyle intervention and how they are prescribed to best support cancer patients and health outcomes is a key element when considering how to prescribe fasting and exercise. Within the recent Physical Activity Guidelines for Americans, a primary recommendation is to break up a prolonged period of sedentary activities by sitting less and moving more [151]. An intervention that focuses on reducing sedentary behaviors may be easier to implement and be more appealing than a strictly supervised exercise prescription. Likewise, the implementation of fasting, where the patient has to limit their food intake for a certain period of time, as opposed to changing the type of food they consume, may be more appealing and easier to adhere to.

### 5.3. Treatment and Diagnosis Considerations

Intervention effects may vary by type of cancer diagnosis and cancer-related treatments. Additional scientific exploration requires investigating the effect by diagnosis given the variability in symptom management (i.e., cancers of the gastrointestinal system). Furthermore, variability in intervention benefits may alter based on pre-existing chronic conditions whereby more vulnerable cancer patients with comorbidities such as diabetes or cardiovascular disease may experience a greater benefit. Cancer-related treatment history is of further consideration as said treatments may negatively alter lifestyle behaviors and increase risk of comorbid conditions providing an opportunity to intervene with a combined fasting and exercise approach.

### 5.4. Cultural Relevancy/Religious Considerations

The mechanisms of fasting in culture are not novel. As mentioned in Table 1, the practice of fasting exists in a variety of religions and cultures. While a few studies have utilized combined fasting and exercise interventions during Ramadan, the impacts of the combined intervention are not clear [40]. To our knowledge, there is a lack of investigations focusing on other types of religious fasting in combination with exercise in our literature search. Therefore, future research needs to consider cultural fasting practices when designing lifestyle intervention studies.

### 5.5. Age Considerations

The impact of fasting and exercise interventions among cancer patients across the lifespan with particular focus on adolescent and young adults, and older cancer survivors warrants investigations [40,79]. The combined impact of exercise and fasting may be particularly impactful for these more vulnerable, understudied populations at high risk for poor cancer outcomes, premature aging, and exacerbated comorbidities [40].

### 5.6. Ongoing Trials

Few ongoing trials are underway examining the impacts of combined exercise and fasting among various populations with only one study focusing on cancer patients (Table 2). The intervention designs were heterogeneous, varying in the number of days per week fasting is incorporated, duration of fasting (i.e., number of fasted hours per day), and modalities of exercise (i.e., aerobic, resistance, or both) [152,153,154,155,156,157,158]. The target populations were diverse, with the majority of the studies targeting a combined young adult and older adult population. Of the studies we identified, most focused on overweight and obese populations with and without comorbidities (e.g., diabetes). Contrary to the design of the studies we have previously reviewed, only one of the identified ongoing trials targeted healthy, young adults, [157] indicating the importance and expansion of this area of research in clinical populations.

The identified ongoing studies are investigating outcomes of interest that will be crucial for understanding the physiological effects and implementing combined fasting and exercise interventions. The majority of the ongoing trials examine the impact of a combined intervention on change in cardiometabolic biomarkers, including insulin sensitivity, insulin resistance, HbA1c, hepatic function, glucose concentrations, lipoprotein lipase, and lipid profiles [152,153,154,155,156,157,158]. Other notable outcomes of interest are changes in body composition, quality of life, cognitive/memory-related measures, physical function, and other patient-reported outcomes. The impact from these ongoing trials will benefit the collective understanding of the effect of combined fasting and exercise across the lifespan in vulnerable cancer populations, and will be important to inform the effectiveness, safety, and feasibility of these interventions in future trials.

## 6. Conclusions

Independently, fasting and exercise are well-tolerated among cancer patients, and while they both induce independent benefits, when combined, their additive or synergistic effects on cardiometabolic, body composition, patient-reported, and cancer-related outcomes are unknown within the cancer population. Many cancer patients experience cancer- and treatment-related side effects, many of which have been demonstrated to be managed, improved, or prevented with energy balance interventions. We are proposing combined fasting and exercise as a potentially viable strategy that may benefit cancer patients and improve cardiometabolic, body composition, patient-reported, and cancer-related outcomes, but much research is required in this area before it is deemed safe and feasible within this population.

## Figures and Tables

**Table 1:** Definitions of different types and concepts of fasting and exercise.

Type/Concept	Definition
Fasting-related	
Intermittent energy restriction	Restricting energy intake to approximately 60–75% below energy requirements for short periods, followed by periods with normal energy intake. One example is the 5:2 diet, consisting of approximately 5 days of eucaloric (a diet that provides the number of calories to maintain your body weight) feeding and approximately 2 days of a very-low-calorie diet per week.
Long-term fasting	Temporarily fasting, typically for a period >72 h.
Short-term fasting	Temporarily fasting, typically for a period between 12 and 72 h. An example of this type of fasting is alternate day fasting.
Time-restricted feeding	Reducing food intake to a set number of hours each day (e.g., eating in a <10 h daily period). One method of time restricted feeding is Prolonged overnight fasting whereby time-restricted feeding occurs overnight.
	(Alternate definition) the practice of consuming ad libitum energy within a restricted window of time and fasting thereafter (upwards of 12–16 h).
Religious fasting	Intermittent fasting exists in some religious practices. These include the Black Fast of Christianity most often practiced during Lent, Varta (Hinduism), Ramadan (Islam), Yom Kippur and other fasts (Judaism), Fast Sunday (Latter-day Saints), Jain (Buddhist) fasting. Religious fasting practices may only require abstinence from certain foods or last for a short period of time and cause negligible effects.
Fasting-mimicking diet	Maintaining a fasting-like state by periodically consuming a very-low-calorie, low-protein diet (not necessarily fasting)
*Exercise-related*	
Exercise	Planned and structured, and repetitive bodily movement in order to improve or maintain physical health outcomes [32].
Physical activity	Any bodily movement produced by skeletal muscles that results in energy expenditure [32].
Physical inactivity	Not performing sufficient amounts of moderate- and vigorous-intensity activity (MVPA), i.e., not meeting specified physical activity guidelines [33].
Sedentary behavior	Any waking behavior characterized by an energy expenditure ≤1.5 metabolic equivalent tasks (METs) while in a sitting, reclining or lying posture [33].

**Table 2:** Ongoing clinical trials with combined fasting and exercise interventions (http://ClinicalTrials.gov, accessed on 1 September 2021).

Identifier	Study Design	Population	Experimental Groups	Intervention Characteristics	Outcomes of Interest
**Cancer Populations**					
NCT04708860 [152]	Single-arm trial	Women ages 18 and older with Metastatic Breast Cancer	Combined POF and exercise	12-week trial POF: Restriction of caloric food/drink after 8 pm, wait 13 h after last meal before eating, fasting 6 days/week Exercise: Moderate-intensity aerobic and strength training, 2 times/week of 30–45 min strength classes, 120 min aerobic activity per week	Primary: Rate of enrollment, rate of adherence to intervention Secondary: Change in metabolic markers, quality of life, and patient-reported outcomes
**Other Populations**					
NCT04004403 [153]	Randomized Clinical Trail	Obese prediabetic adults ages 18–64 with NAFLD	(1) ADF (2) Exercise (ad libitum fed) (3) ADF + exercise (4) Control (ad libitum fed, no exercise)	24-week trial ADF: Fast day: 25% energy intake (~500 kcal), Feed day: ad libitum fed Exercise: Aerobic exercise training, 5 sessions/week	Primary: Change in hepatic steatosis, body weight Secondary: Change in hepatokine profile, hepatic insulin sensitivity, insulin resistance, HbA1c, and other metabolic disease risk factors
NCT04131647 [154]	Randomized Clinical Trail	Overweight and obese older adult (ages 50–70) veterans	(1) Weight Maintenance Only (2) Weight maintenance + IF	24-week program Weight Maintenance Program: Nutrition advice, walking, and resistance training, 12-week program IF + Exercise: One day of IF per week, consisting of 2 small meals/day; the combined program continues for 24-week program, following completion of the 12-week weight maintenance program; walking and resistance exercises	Primary: Change in body weight Secondary: Change in gait speed, body fat, lipoprotein lipase
NCT04585581 [155]	Randomized Clinical Trail	Overweight women getting pregnant in next 6 months	(1) TRE + HIIT Exercise (2) Standard Care	Duration of pregnancy (min. 28 weeks) TRE + Exercise: Minimum of 14 h/day HIIT exercise: 2–3 days/week	Primary: Plasma glucose concentration at gestational week 28 Secondary: Maternal and offspring cardiometabolic health measures
NCT04768725 [156]	Randomized Clinical Trail	Obese, postmenopausal women ages 45–59, sedentary lifestyle	(1) IF (2) IF + Physical-Cognitive Exergaming Program (3) Control	12-week trial IF: Self-selected diet with 25–75% of estimated baseline energy requirements for 2 days/week (fast day) along with ad libitum for 5 days/week (feed day) Exercise: Moderate-Vigorous intensity [60–70% of heart rate maximum for aerobic and 60–70% of 1 repetition maximum, 8–12 repetitions/set, 3 sets of each exercise for resistance exercise, 60 min/session, 3 sessions/week (36 total sessions)]	Primary: Change in cognitive (memory, logic, brain-derived neurotropic factor) outcomes Secondary: Change in additional cognitive and functional measures, biomarkers, physical function
NCT04834687 [157]	Randomized Clinical Trail	Healthy 17–24-year-old young adults	(1) Exercise-Only (2) TRE (3) Combined TRE + Exercise (4) Control	Trial length not reported Exercise: Aerobic rope-skipping, 3 days/week, 90 min/session TRE: 14 h fast, 10 h eating window, high-fiber diet	Primary: Change in body weight Secondary: Change in cardiovascular metabolic markers, executive function, intestinal flora
NCT04978376 [158]	Non-Randomized Clinical Trial	Overweight, older adults 50–70 years old with pre-diabetes	(1) TRE-only (2) TRE + Endurance Exercise (3) TRE with Resistance Exercise (4) Control	10-week trial TRE is restricted eating with ad libitum eating between 12:00–20:00 Endurance Exercise: 3–5 days/week of supervised exercise Resistance Exercise: 3–5 days/week of supervised exercise	Primary: Change in body weight Secondary: Change in body composition, insulin, glucose, and HbA1c

Abbreviations: POF = Prolonged Overnight Fasting; NAFLD = Non-Alcoholic Fatty Liver Disease; ADF = Alternate-Day Fasting; IF = Intermittent Fasting; TRE = Time-Restricted Eating; HIIT = High-Intensity Interval Training.

## Data Availability

Not applicable.

## References

[1] Buchinger O. (1959). 40 Years of fasting therapy. Hippokrates.

[2] Cespedes Feliciano E.M., Chen W.Y., Bradshaw P.T., Prado C.M., Alexeeff S., Albers K.B., Castillo A.L., Caan B.J. (2019). Adipose tissue distribution and cardiovascular disease risk among breast cancer survivors. J. Clin. Oncol..

[3] Keating N.L., O’Malley A.J., Freedland S.J., Smith M.R. (2010). Diabetes and cardiovascular disease during androgen deprivation therapy: Observational study of veterans with prostate cancer. J. Natl. Cancer Inst..

[4] Felicetti F., Fortunati N., Brignardello E. (2018). Cancer survivors: An expanding population with an increased cardiometabolic risk. Diabetes Res. Clin. Pract..

[5] Gupta D., Lee Chuy K., Yang J.C., Bates M., Lombardo M., Steingart R.M. (2018). Cardiovascular and metabolic effects of androgen-deprivation therapy for prostate cancer. J. Oncol. Pract..

[6] Corremans R., Adao R., De Keulenaer G.W., Leite-Moreira A.F., Bras-Silva C. (2019). Update on pathophysiology and preventive strategies of anthracycline-induced cardiotoxicity. Clin. Exp. Pharmacol. Physiol..

[7] Cardinale D., Iacopo F., Cipolla C.M. (2020). Cardiotoxicity of anthracyclines. Front. Cardiovasc. Med..

[8] Wilson R.L., Shannon T., Calton E., Galvao D.A., Taaffe D.R., Hart N.H., Lyons-Wall P., Newton R.U. (2020). Efficacy of a weight loss program prior to robot assisted radical prostatectomy in overweight and obese men with prostate cancer. Surg. Oncol..

[9] Newton R.U., Jeffery E., Galvao D.A., Peddle-McIntyre C.J., Spry N., Joseph D., Denham J.W., Taaffe D.R. (2018). Body composition, fatigue and exercise in patients with prostate cancer undergoing androgen-deprivation therapy. BJU Int..

[10] Baker A.M., Smith K.C., Coa K.I., Helzlsouer K.J., Caulfield L.E., Peairs K.S., Shockney L.D., Klassen A.C. (2015). Clinical care providers’ perspectives on body size and weight management among long-term cancer survivors. Integr. Cancer Ther..

[11] Thomas R.J., Holm M., Williams M., Bowman E., Bellamy P., Andreyev J., Maher J. (2013). Lifestyle factors correlate with the risk of late pelvic symptoms after prostatic radiotherapy. Clin. Oncol..

[12] Ryan A.M., Power D.G., Daly L., Cushen S.J., Ní Bhuachalla Ē., Prado C.M. (2016). Cancer-associated malnutrition, cachexia and sarcopenia: The skeleton in the hospital closet 40 years later. Proc. Nutr. Soc..

[13] Burden S., Jones D.J., Sremanakova J., Sowerbutts A.M., Lal S., Pilling M., Todd C. (2019). Dietary interventions for adult cancer survivors. Cochrane Database Syst. Rev..

[14] Christensen J.F., Simonsen C., Hojman P. (2018). Exercise training in cancer control and treatment. Compr. Physiol..

[15] Cathcart P., Craddock C., Stebbing J. (2017). Fasting: Starving cancer. Lancet Oncol..

[16] Bauersfeld S.P., Kessler C.S., Wischnewsky M., Jaensch A., Steckhan N., Stange R., Kunz B., Bruckner B., Sehouli J., Michalsen A. (2018). The effects of short-term fasting on quality of life and tolerance to chemotherapy in patients with breast and ovarian cancer: A randomized cross-over pilot study. BMC Cancer.

[17] de Groot S., Lugtenberg R.T., Cohen D., Welters M.J.P., Ehsan I., Vreeswijk M.P.G., Smit V.T.H.B.M., de Graaf H., Heijns J.B., Portielje J.E.A. (2020). Fasting mimicking diet as an adjunct to neoadjuvant chemotherapy for breast cancer in the multicentre randomized phase 2 DIRECT trial. Nat. Commun..

[18] Dorff T.B., Groshen S., Garcia A., Shah M., Tsao-Wei D., Pham H., Cheng C.W., Brandhorst S., Cohen P., Wei M. (2016). Safety and feasibility of fasting in combination with platinum-based chemotherapy. BMC Cancer.

[19] de Groot S., Vreeswijk M.P., Welters M.J., Gravesteijn G., Boei J.J., Jochems A., Houtsma D., Putter H., van der Hoeven J.J., Nortier J.W. (2015). The effects of short-term fasting on tolerance to (neo) adjuvant chemotherapy in HER2-negative breast cancer patients: A randomized pilot study. BMC Cancer.

[20] Safdie F.M., Dorff T., Quinn D., Fontana L., Wei M., Lee C., Cohen P., Longo V.D. (2009). Fasting and cancer treatment in humans: A case series report. Aging.

[21] De Groot S., Pijl H., van der Hoeven J.J., Kroep J.R. (2019). Effects of short-term fasting on cancer treatment. J. Exp. Clin. Cancer Res..

[22] Doyle C., Kushi L.H., Byers T., Courneya K.S., Demark-Wahnefried W., Grant B., McTiernan A., Rock C.L., Thompson C., Gansler T. (2006). Nutrition and physical activity during and after cancer treatment: An American Cancer Society guide for informed choices. CA Cancer J. Clin..

[23] Wei M., Brandhorst S., Shelehchi M., Mirzaei H., Cheng C.W., Budniak J., Groshen S., Mack W.J., Guen E., Di Biase S. (2017). Fasting-mimicking diet and markers/risk factors for aging, diabetes, cancer, and cardiovascular disease. Sci. Transl. Med..

[24] Di Biase S., Lee C., Brandhorst S., Manes B., Buono R., Cheng C.W., Cacciottolo M., Martin-Montalvo A., de Cabo R., Wei M. (2016). Fasting-Mimicking Diet Reduces HO-1 to Promote T Cell-Mediated Tumor Cytotoxicity. Cancer Cell.

[25] Campbell K.L., Winters-Stone K.M., Wiskemann J., May A.M., Schwartz A.L., Courneya K.S., Zucker D.S., Matthews C.E., Ligibel J.A., Gerber L.H. (2019). Exercise Guidelines for Cancer Survivors: Consensus Statement from International Multidisciplinary Roundtable. Med. Sci. Sports Exerc..

[26] Schmitz K.H. (2020). Exercise Oncology: Prescribing Physical Activity before and after a Cancer Diagnosis.

[27] Bland K.A., Zadravec K., Landry T., Weller S., Meyers L., Campbell K.L. (2019). Impact of exercise on chemotherapy completion rate: A systematic review of the evidence and recommendations for future exercise oncology research. Crit. Rev. Oncol. Hematol..

[28] Morielli A.R., Courneya K.S. (2020). Effects of exercise on cancer treatment completion and efficacy. Exercise Oncology: Prescribing Physical Activity before and after a Cancer Diagnosis.

[29] Courneya K.S., Segal R.J., McKenzie D.C., Dong H., Gelmon K., Friedenreich C.M., Yasui Y., Reid R.D., Crawford J.J., Mackey J.R. (2014). Effects of exercise during adjuvant chemotherapy on breast cancer outcomes. Med. Sci. Sports Exerc..

[30] Rief H., Bruckner T., Schlampp I., Bostel T., Welzel T., Debus J., Förster R. (2016). Resistance training concomitant to radiotherapy of spinal bone metastases—Survival and prognostic factors of a randomized trial. Radiat. Oncol..

[31] Wiskemann J., Kleindienst N., Kuehl R., Dreger P., Schwerdtfeger R., Bohus M. (2015). Effects of physical exercise on survival after allogeneic stem cell transplantation. Int. J. Cancer.

[32] Caspersen C.J., Powell K.E., Christenson G.M. (1985). Physical activity, exercise, and physical fitness: Definitions and distinctions for health-related research. Public Health Rep..

[33] Van der Ploeg H.P., Hillsdon M. (2017). Is sedentary behaviour just physical inactivity by another name?. Int. J. Behav. Nutr. Phys. Act..

[34] Jaspers R.T., Zillikens M.C., Friesema E.C., delli Paoli G., Bloch W., Uitterlinden A.G., Goglia F., Lanni A., de Lange P. (2017). Exercise, fasting, and mimetics: Toward beneficial combinations?. FASEB J..

[35] Wallis G.A., Gonzalez J.T. (2019). Is exercise best served on an empty stomach?. Proc. Nutr. Soc..

[36] Hansen D., De Strijcker D., Calders P. (2017). Impact of endurance exercise training in the fasted state on muscle biochemistry and metabolism in healthy subjects: Can these effects be of particular clinical benefit to type 2 diabetes mellitus and insulin-resistant patients?. Sports Med..

[37] Albrecht T.A., Anderson J.G., Jones R., Bourguignon C., Taylor A.G. (2012). A complementary care study combining flaxseed oil, caffeine, fasting, and exercise in women diagnosed with advanced ovarian cancer: Findings from a case study. Holist. Nurs. Pract..

[38] Rui L. (2014). Energy metabolism in the liver. Compr. Physiol..

[39] Stote K.S., Baer D.J., Spears K., Paul D.R., Harris G.K., Rumpler W.V., Strycula P., Najjar S.S., Ferrucci L., Ingram D.K. (2007). A controlled trial of reduced meal frequency without caloric restriction in healthy, normal-weight, middle-aged adults. Am. J. Clin. Nutr..

[40] Keenan S., Cooke M.B., Belski R. (2020). The effects of intermittent fasting combined with resistance training on lean body mass: A systematic review of human studies. Nutrients.

[41] Donnelly R.P., Finlay D.K. (2015). Glucose, glycolysis and lymphocyte responses. Mol. Immunol..

[42] Coyle E.F., Jeukendrup A.E., Wagenmakers A.J., Saris W.H. (1997). Fatty acid oxidation is directly regulated by carbohydrate metabolism during exercise. Am. J. Physiol..

[43] De Bock K., Richter E.A., Russell A.P., Eijnde B.O., Derave W., Ramaekers M., Koninckx E., Leger B., Verhaeghe J., Hespel P. (2005). Exercise in the fasted state facilitates fibre type-specific intramyocellular lipid breakdown and stimulates glycogen resynthesis in humans. J. Physiol..

[44] Thomas D.E., Brotherhood J.R., Brand J.C. (1991). Carbohydrate feeding before exercise: Effect of glycemic index. Int. J. Sports Med..

[45] Vieira A.F., Costa R.R., Macedo R.C., Coconcelli L., Kruel L.F. (2016). Effects of aerobic exercise performed in fasted v. fed state on fat and carbohydrate metabolism in adults: A systematic review and meta-analysis. Br. J. Nutr..

[46] Siegel R.L., Miller K.D., Jemal A. (2020). Cancer statistics, 2020. CA Cancer J. Clin..

[47] Weroha S.J., Haluska P. (2012). The insulin-like growth factor system in cancer. Endocrinol. Metab. Clin. N. Am..

[48] Pollak M.N., Schernhammer E.S., Hankinson S.E. (2004). Insulin-like growth factors and neoplasia. Nat. Rev. Cancer.

[49] Calle E.E., Rodriguez C., Walker-Thurmond K., Thun M.J. (2003). Overweight, obesity, and mortality from cancer in a prospectively studied cohort of U.S. adults. N. Engl. J. Med..

[50] Zorn S., Ehret J., Schäuble R., Rautenberg B., Ihorst G., Bertz H., Urbain P., Raynor A. (2020). Impact of modified short-term fasting and its combination with a fasting supportive diet during chemotherapy on the incidence and severity of chemotherapy-induced toxicities in cancer patients-a controlled cross-over pilot study. BMC Cancer.

[51] Marinac C.R., Nelson S.H., Breen C.I., Hartman S.J., Natarajan L., Pierce J.P., Flatt S.W., Sears D.D., Patterson R.E. (2016). Prolonged nightly fasting and breast cancer prognosis. JAMA Oncol..

[52] Ferroni P., Riondino S., Laudisi A., Portarena I., Formica V., Alessandroni J., D’Alessandro R., Orlandi A., Costarelli L., Cavaliere F. (2016). Pretreatment insulin levels as a prognostic factor for breast cancer progression. Oncologist.

[53] Duggan C., Wang C.Y., Neuhouser M.L., Xiao L., Smith A.W., Reding K.W., Baumgartner R.N., Baumgartner K.B., Bernstein L., Ballard-Barbash R. (2013). Associations of insulin-like growth factor and insulin-like growth factor binding protein-3 with mortality in women with breast cancer. Int. J. Cancer.

[54] Meyerhardt J.A., Catalano P.J., Haller D.G., Mayer R.J., Macdonald J.S., Benson A.B., Fuchs C.S. (2003). Impact of diabetes mellitus on outcomes in patients with colon cancer. J. Clin. Oncol..

[55] Derr R.L., Ye X., Islas M.U., Desideri S., Saudek C.D., Grossman S.A. (2009). Association between hyperglycemia and survival in patients with newly diagnosed glioblastoma. J. Clin. Oncol..

[56] Kang D.W., Fairey A.S., Boule N.G., Field C.J., Courneya K.S. (2017). Effects of exercise on insulin, IGF axis, adipocytokines, and inflammatory markers in breast cancer survivors: A systematic review and meta-analysis. Cancer Epidemiol. Biomark. Prev..

[57] Wang Y., Jin B., Paxton R.J., Yang W., Wang X., Jiao Y., Yu C., Chen X. (2020). The effects of exercise on insulin, glucose, IGF-axis and CRP in cancer survivors: Meta-analysis and meta-regression of randomised controlled trials. Eur. J. Cancer Care.

[58] Arcidiacono B., Iiritano S., Nocera A., Possidente K., Nevolo M.T., Ventura V., Foti D., Chiefari E., Brunetti A. (2012). Insulin resistance and cancer risk: An overview of the pathogenetic mechanisms. Exp. Diabetes Res..

[59] Butler L.M., Perone Y., Dehairs J., Lupien L.E., de Laat V., Talebi A., Loda M., Kinlaw W.B., Swinnen J.V. (2020). Lipids and cancer: Emerging roles in pathogenesis, diagnosis and therapeutic intervention. Adv. Drug Deliv. Rev..

[60] Goodyear L.J., Kahn B.B. (1998). Exercise, glucose transport, and insulin sensitivity. Annu. Rev. Med..

[61] Pesta D.H., Goncalves R.L.S., Madiraju A.K., Strasser B., Sparks L.M. (2017). Resistance training to improve type 2 diabetes: Working toward a prescription for the future. Nutr. Metab..

[62] Keshel T.E., Coker R.H. (2015). Exercise Training and Insulin Resistance: A current review. J. Obes. Weight Loss Ther..

[63] Horowitz J.F., Klein S. (2000). Lipid metabolism during endurance exercise. Am. J. Clin. Nutr..

[64] Gordon B., Chen S., Durstine J.L. (2014). The effects of exercise training on the traditional lipid profile and beyond. Curr. Sports Med. Rep..

[65] Mann S., Beedie C., Jimenez A. (2014). Differential effects of aerobic exercise, resistance training and combined exercise modalities on cholesterol and the lipid profile: Review, synthesis and recommendations. Sports Med..

[66] Noland R.C., Bouchard C. (2015). Chapter Three—Exercise and Regulation of Lipid Metabolism. Progress in Molecular Biology and Translational Science.

[67] Haxhi J., Scotto di Palumbo A., Sacchetti M. (2013). Exercising for metabolic control: Is timing important?. Ann. Nutr. Metab..

[68] Bhutani S., Klempel M.C., Kroeger C.M., Trepanowski J.F., Varady K.A. (2013). Alternate day fasting and endurance exercise combine to reduce body weight and favorably alter plasma lipids in obese humans. Obesity.

[69] Cho A.R., Moon J.Y., Kim S., An K.Y., Oh M., Jeon J.Y., Jung D.H., Choi M.H., Lee J.W. (2019). Effects of alternate day fasting and exercise on cholesterol metabolism in overweight or obese adults: A pilot randomized controlled trial. Metabolism.

[70] Galvao D.A., Taaffe D.R., Spry N., Joseph D., Newton R.U. (2011). Acute versus chronic exposure to androgen suppression for prostate cancer: Impact on the exercise response. J. Urol..

[71] Kelley D.E., Goodpaster B.H. (2001). Skeletal muscle triglyceride. An aspect of regional adiposity and insulin resistance. Diabetes Care.

[72] Collins K.H., Herzog W., MacDonald G.Z., Reimer R.A., Rios J.L., Smith I.C., Zernicke R.F., Hart D.A. (2018). Obesity, metabolic syndrome, and musculoskeletal disease: Common inflammatory pathways suggest a central role for loss of muscle integrity. Front. Physiol..

[73] Cheung A.S., Hoermann R., Dupuis P., Joon D.L., Zajac J.D., Grossmann M. (2016). Relationships between insulin resistance and frailty with body composition and testosterone in men undergoing androgen deprivation therapy for prostate cancer. Eur. J. Endocrinol..

[74] van Loon L.J., Koopman R., Stegen J.H., Wagenmakers A.J., Keizer H.A., Saris W.H. (2003). Intramyocellular lipids form an important substrate source during moderate intensity exercise in endurance-trained males in a fasted state. J. Physiol..

[75] Qu H.-Q., Li Q., Rentfro A.R., Fisher-Hoch S.P., McCormick J.B. (2011). The definition of insulin resistance using HOMA-IR for Americans of Mexican descent using machine learning. PLoS ONE.

[76] Tang Q., Li X., Song P., Xu L. (2015). Optimal cut-off values for the homeostasis model assessment of insulin resistance (HOMA-IR) and pre-diabetes screening: Developments in research and prospects for the future. Drug Discov. Ther..

[77] Yamada C., Mitsuhashi T., Hiratsuka N., Inabe F., Araida N., Takahashi E. (2011). Optimal reference interval for homeostasis model assessment of insulin resistance in a Japanese population. J Diabetes Investig..

[78] Lee C.H., Shih A.Z., Woo Y.C., Fong C.H., Leung O.Y., Janus E., Cheung B.M., Lam K.S. (2016). Optimal cut-offs of homeostasis model assessment of insulin resistance (HOMA-IR) to identify dysglycemia and type 2 diabetes mellitus: A 15-year prospective study in Chinese. PLoS ONE.

[79] Van Proeyen K., Szlufcik K., Nielens H., Pelgrim K., Deldicque L., Hesselink M., Van Veldhoven P.P., Hespel P. (2010). Training in the fasted state improves glucose tolerance during fat-rich diet. J. Physiol..

[80] Cespedes Feliciano E.M., Kroenke C.H., Caan B.J. (2018). The obesity paradox in cancer: How important is muscle?. Annu. Rev. Nutr..

[81] Galvao D.A., Spry N.A., Taaffe D.R., Newton R.U., Stanley J., Shannon T., Rowling C., Prince R. (2008). Changes in muscle, fat and bone mass after 36 weeks of maximal androgen blockade for prostate cancer. BJU Int..

[82] Lee K., Kruper L., Dieli-Conwright C.M., Mortimer J.E. (2019). The impact of obesity on breast cancer diagnosis and treatment. Curr. Oncol. Rep..

[83] Dickerman B.A., Torfadottir J.E., Valdimarsdottir U.A., Giovannucci E., Wilson K.M., Aspelund T., Tryggvadottir L., Sigurdardottir L.G., Harris T.B., Launer L.J. (2019). Body fat distribution on computed tomography imaging and prostate cancer risk and mortality in the AGES-Reykjavik study. Cancer.

[84] De Nardi P., Salandini M., Chiari D., Pecorelli N., Cristel G., Damascelli A., Ronzoni M., Massimino L., De Cobelli F., Braga M. (2020). Changes in body composition during neoadjuvant therapy can affect prognosis in rectal cancer patients: An exploratory study. Curr. Probl. Cancer.

[85] Michalsen A., Li C. (2013). Fasting therapy for treating and preventing disease—Current state of evidence. Forsch. Komplement..

[86] Köppel M., Mathis K., Schmitz K.H., Wiskemann J. (2021). Muscle hypertrophy in cancer patients and survivors via strength training. A meta-analysis and meta-regression. Crit. Rev. Oncol. Hematol..

[87] Haseen F., Murray L.J., Cardwell C.R., O’Sullivan J.M., Cantwell M.M. (2010). The effect of androgen deprivation therapy on body composition in men with prostate cancer: Systematic review and meta-analysis. J. Cancer Surviv..

[88] Chang D., Joseph D.J., Ebert M.A., Galvão D.A., Taaffe D.R., Denham J.W., Newton R.U., Spry N.A. (2014). Effect of androgen deprivation therapy on muscle attenuation in men with prostate cancer. J. Med. Imaging Radiat. Oncol..

[89] Lopez P., Taaffe D.R., Newton R.U., Galvão D.A. (2021). Resistance exercise dosage in men with prostate cancer: Systematic review, meta-analysis, and meta-regression. Med. Sci. Sports Exerc..

[90] Zdravkovic A., Hasenöhrl T., Palma S., Crevenna R. (2020). Effects of resistance exercise in prostate cancer patients: A systematic review update as of March 2020. Wien. Klin. Wochenschr..

[91] McTiernan A. (2005). Obesity and cancer: The risks, science, and potential management strategies. Oncology.

[92] Irwin M.L., Alvarez-Reeves M., Cadmus L., Mierzejewski E., Mayne S.T., Yu H., Chung G.G., Jones B., Knobf M.T., DiPietro L. (2009). Exercise improves body fat, lean mass, and bone mass in breast cancer survivors. Obesity.

[93] Guinan E.M., Connolly E.M., Hussey J. (2013). Exercise training in breast cancer survivors: A review of trials examining anthropometric and obesity-related biomarkers of breast cancer risk. Phys. Ther. Rev..

[94] Thomas G.A., Cartmel B., Harrigan M., Fiellin M., Capozza S., Zhou Y., Ercolano E., Gross C.P., Hershman D., Ligibel J. (2017). The effect of exercise on body composition and bone mineral density in breast cancer survivors taking aromatase inhibitors. Obesity.

[95] Brown J.C., Zemel B.S., Troxel A.B., Rickels M.R., Damjanov N., Ky B., Rhim A.D., Rustgi A.K., Courneya K.S., Schmitz K.H. (2017). Dose–response effects of aerobic exercise on body composition among colon cancer survivors: A randomised controlled trial. Br. J. Cancer.

[96] Dieli-Conwright C.M., Mortimer J.E., Schroeder E.T., Courneya K., Demark-Wahnefried W., Buchanan T.A., Tripathy D., Bernstein L. (2018). Effects of aerobic and resistance exercise on metabolic syndrome, sarcopenic obesity, and circulating biomarkers in overweight or obese survivors of breast cancer: A randomized controlled trial. J. Clin. Oncol..

[97] Reeves M.M., Terranova C.O., Eakin E.G., Demark-Wahnefried W. (2014). Weight loss intervention trials in women with breast cancer: A systematic review. Obes. Rev..

[98] LeVasseur N., Cheng W., Mazzarello S., Clemons M., Vandermeer L., Jones L., Joy A.A., Barbeau P., Wolfe D., Ahmadzai N. (2021). Optimising weight-loss interventions in cancer patients—A systematic review and network meta-analysis. PLoS ONE.

[99] Lee J. (2021). The effects of resistance training on muscular strength and hypertrophy in elderly cancer patients: A systematic review and meta-analysis. J. Sport Health Sci..

[100] Deng T., Lyon C.J., Bergin S., Caligiuri M.A., Hsueh W.A. (2016). Obesity, inflammation, and cancer. Annu. Rev. Pathol..

[101] Schoenfeld B.J., Aragon A.A., Wilborn C.D., Krieger J.W., Sonmez G.T. (2014). Body composition changes associated with fasted versus non-fasted aerobic exercise. J. Int. Soc. Sports Nutr..

[102] Stroup S.P., Cullen J., Auge B.K., L’Esperance J.O., Kang S.K. (2007). Effect of obesity on prostate-specific antigen recurrence after radiation therapy for localized prostate cancer as measured by the 2006 Radiation Therapy Oncology Group-American Society for Therapeutic Radiation and Oncology (RTOG-ASTRO) Phoenix consensus definition. Cancer.

[103] Fearon K., Strasser F., Anker S.D., Bosaeus I., Bruera E., Fainsinger R.L., Jatoi A., Loprinzi C., MacDonald N., Mantovani G. (2011). Definition and classification of cancer cachexia: An international consensus. Lancet Oncol..

[104] Hanson E.D., Nelson A.R., West D.W., Violet J.A., O’keefe L., Phillips S.M., Hayes A. (2017). Attenuation of resting but not load-mediated protein synthesis in prostate cancer patients on androgen deprivation. J. Clin. Endocrinol. Metab..

[105] Cermak N.M., Res P.T., de Groot L.C., Saris W.H., van Loon L.J. (2012). Protein supplementation augments the adaptive response of skeletal muscle to resistance-type exercise training: A meta-analysis. Am. J. Clin. Nutr..

[106] Slater G.J., Dieter B.P., Marsh D.J., Helms E.R., Shaw G., Iraki J. (2019). Is an Energy Surplus Required to Maximize Skeletal Muscle Hypertrophy Associated With Resistance Training. Front. Nutr..

[107] Murphy C., Koehler K. (2020). Caloric restriction induces anabolic resistance to resistance exercise. Eur. J. Appl. Physiol..

[108] Tinsley G.M., Moore M.L., Graybeal A.J., Paoli A., Kim Y., Gonzales J.U., Harry J.R., VanDusseldorp T.A., Kennedy D.N., Cruz M.R. (2019). Time-restricted feeding plus resistance training in active females: A randomized trial. Am. J. Clin. Nutr..

[109] Stark L., Tofthagen C., Visovsky C., McMillan S.C. (2012). The symptom experience of patients with cancer. J. Hosp. Palliat. Nurs..

[110] Weber D., O’Brien K. (2017). Cancer and cancer-related fatigue and the interrelationships with depression, stress, and inflammation. J. Evid. Based Complement. Altern. Med..

[111] Theobald D.E. (2004). Cancer pain, fatigue, distress, and insomnia in cancer patients. Clin. Cornerstone.

[112] Buffart L.M., Kalter J., Sweegers M.G., Courneya K.S., Newton R.U., Aaronson N.K., Jacobsen P.B., May A.M., Galvão D.A., Chinapaw M.J. (2017). Effects and moderators of exercise on quality of life and physical function in patients with cancer: An individual patient data meta-analysis of 34 RCTs. Cancer Treat. Rev..

[113] Mishra S.I., Scherer R.W., Geigle P.M., Berlanstein D.R., Topaloglu O., Gotay C.C., Snyder C. (2012). Exercise interventions on health-related quality of life for cancer survivors. Cochrane Database Syst. Rev..

[114] Kessels E., Husson O., Van der Feltz-Cornelis C.M. (2018). The effect of exercise on cancer-related fatigue in cancer survivors: A systematic review and meta-analysis. Neuropsychiatr. Dis. Treat..

[115] Mustian K.M., Alfano C.M., Heckler C., Kleckner A.S., Kleckner I.R., Leach C.R., Mohr D., Palesh O.G., Peppone L.J., Piper B.F. (2017). Comparison of pharmaceutical, psychological, and exercise treatments for cancer-related fatigue: A meta-analysis. JAMA Oncol..

[116] Brown J.C., Huedo-Medina T.B., Pescatello L.S., Ryan S.M., Pescatello S.M., Moker E., LaCroix J.M., Ferrer R.A., Johnson B.T. (2012). The efficacy of exercise in reducing depressive symptoms among cancer survivors: A meta-analysis. PLoS ONE.

[117] Kang D.W., Fairey A.S., Boule N.G., Field C.J., Courneya K.S. (2019). Exercise duRing Active Surveillance for prostatE cancer-the ERASE trial: A study protocol of a phase II randomised controlled trial. BMJ Open.

[118] Cartmel B., Hughes M., Ercolano E.A., Gottlieb L., Li F., Zhou Y., Harrigan M., Ligibel J.A., von Gruenigen V.E., Gogoi R. (2021). Randomized trial of exercise on depressive symptomatology and brain derived neurotrophic factor (BDNF) in ovarian cancer survivors: The Women’s Activity and Lifestyle Study in Connecticut (WALC). Gynecol. Oncol..

[119] i Ferrer B.-C.S., van Roekel E., Lynch B.M. (2018). The role of physical activity in managing fatigue in cancer survivors. Curr. Nutr. Rep..

[120] Bourke L., Gilbert S., Hooper R., Steed L.A., Joshi M., Catto J.W., Saxton J.M., Rosario D.J. (2014). Lifestyle changes for improving disease-specific quality of life in sedentary men on long-term androgen-deprivation therapy for advanced prostate cancer: A randomised controlled trial. Eur. Urol..

[121] Kohler L.N., Garcia D.O., Harris R.B., Oren E., Roe D.J., Jacobs E.T. (2016). Adherence to diet and physical activity cancer prevention guidelines and cancer outcomes: A systematic review. Cancer Epidemiol. Biomark. Prev..

[122] Warburg O. (1956). On the origin of cancer cells. Science.

[123] Romijn J., Godfried M., Hommes M., Endert E., Sauerwein H. (1990). Decreased glucose oxidation during short-term starvation. Metabolism.

[124] Pedersen L., Christensen J.F., Hojman P. (2015). Effects of exercise on tumor physiology and metabolism. Cancer J..

[125] Fontana L., Weiss E.P., Villareal D.T., Klein S., Holloszy J.O. (2008). Long-term effects of calorie or protein restriction on serum IGF-1 and IGFBP-3 concentration in humans. Aging Cell.

[126] Renehan A.G., Zwahlen M., Minder C., T O’Dwyer S., Shalet S.M., Egger M. (2004). Insulin-like growth factor (IGF)-I, IGF binding protein-3, and cancer risk: Systematic review and meta-regression analysis. Lancet.

[127] Walford R.L., Mock D., Verdery R., MacCallum T. (2002). Calorie restriction in biosphere 2: Alterations in physiologic, hematologic, hormonal, and biochemical parameters in humans restricted for a 2-year period. J. Gerontol. Ser. A Biol. Sci. Med. Sci..

[128] Raffaghello L., Lee C., Safdie F.M., Wei M., Madia F., Bianchi G., Longo V.D. (2008). Starvation-dependent differential stress resistance protects normal but not cancer cells against high-dose chemotherapy. Proc. Natl. Acad. Sci. USA.

[129] Lee C., Raffaghello L., Brandhorst S., Safdie F.M., Bianchi G., Martin-Montalvo A., Pistoia V., Wei M., Hwang S., Merlino A. (2012). Fasting cycles retard growth of tumors and sensitize a range of cancer cell types to chemotherapy. Sci. Transl. Med..

[130] Safdie F., Brandhorst S., Wei M., Wang W., Lee C., Hwang S., Conti P.S., Chen T.C., Longo V.D. (2012). Fasting enhances the response of glioma to chemo- and radiotherapy. PLoS ONE.

[131] Laviano A., Rossi Fanelli F. (2012). Toxicity in chemotherapy—When less is more. N. Engl. J. Med..

[132] Longo V.D., Mattson M.P. (2014). Fasting: Molecular mechanisms and clinical applications. Cell Metab..

[133] Fontana L., Partridge L., Longo V.D. (2010). Extending healthy life span—From yeast to humans. Science.

[134] Bishop N.A., Guarente L. (2007). Genetic links between diet and lifespan: Shared mechanisms from yeast to humans. Nat. Rev. Genet..

[135] Cheng C.W., Adams G.B., Perin L., Wei M., Zhou X., Lam B.S., Da Sacco S., Mirisola M., Quinn D.I., Dorff T.B. (2014). Prolonged fasting reduces IGF-1/PKA to promote hematopoietic-stem-cell-based regeneration and reverse immunosuppression. Cell Stem Cell.

[136] Sun P., Wang H., He Z., Chen X., Wu Q., Chen W., Sun Z., Weng M., Zhu M., Ma D. (2017). Fasting inhibits colorectal cancer growth by reducing M2 polarization of tumor-associated macrophages. Oncotarget.

[137] Koelwyn G.J., Zhuang X., Tammela T., Schietinger A., Jones L.W. (2020). Exercise and immunometabolic regulation in cancer. Nat. Metab..

[138] Hojman P., Gehl J., Christensen J.F., Pedersen B.K. (2018). Molecular Mechanisms Linking Exercise to Cancer Prevention and Treatment. Cell Metab..

[139] Pedersen L., Idorn M., Olofsson G.H., Lauenborg B., Nookaew I., Hansen R.H., Johannesen H.H., Becker J.C., Pedersen K.S., Dethlefsen C. (2016). Voluntary Running Suppresses Tumor Growth through Epinephrine- and IL-6-Dependent NK Cell Mobilization and Redistribution. Cell Metab..

[140] Idorn M., Hojman P. (2016). Exercise-Dependent Regulation of NK Cells in Cancer Protection. Trends Mol. Med..

[141] Betof A.S., Lascola C.D., Weitzel D., Landon C., Scarbrough P.M., Devi G.R., Palmer G., Jones L.W., Dewhirst M.W. (2015). Modulation of murine breast tumor vascularity, hypoxia, and chemotherapeutic response by exercise. JNCI J. Natl. Cancer Inst..

[142] Morrell M.B.G., Alvarez-Florez C., Zhang A., Kleinerman E.S., Savage H., Marmonti E., Park M., Shaw A., Schadler K.L. (2019). Vascular modulation through exercise improves chemotherapy efficacy in Ewing sarcoma. Pediatr. Blood Cancer.

[143] Yang L., Morielli A.R., Heer E., Kirkham A.A., Cheung W.Y., Usmani N., Friedenreich C.M., Courneya K.S. (2021). Effects of Exercise on Cancer Treatment Efficacy: A Systematic Review of Preclinical and Clinical Studies. Cancer Res..

[144] Pin F., Couch M.E., Bonetto A. (2018). Preservation of muscle mass as a strategy to reduce the toxic effects of cancer chemotherapy on body composition. Curr. Opin. Support. Palliat. Care.

[145] Cespedes Feliciano E.M., Chen W.Y., Lee V., Albers K.B., Prado C.M., Alexeeff S., Xiao J., Shachar S.S., Caan B.J. (2020). Body composition, adherence to anthracycline and taxane-based chemotherapy, and survival after nonmetastatic breast cancer. JAMA Oncol..

[146] Prado C.M., Lieffers J.R., McCargar L.J., Reiman T., Sawyer M.B., Martin L., Baracos V.E. (2008). Prevalence and clinical implications of sarcopenic obesity in patients with solid tumours of the respiratory and gastrointestinal tracts: A population-based study. Lancet Oncol..

[147] Dupertuis Y.M., Meguid M.M., Pichard C. (2007). Colon cancer therapy: New perspectives of nutritional manipulations using polyunsaturated fatty acids. Curr. Opin. Clin. Nutr. Metab. Care.

[148] Scheim D.E. (2009). Cytotoxicity of unsaturated fatty acids in fresh human tumor explants: Concentration thresholds and implications for clinical efficacy. Lipids Health Dis..

[149] Comba A., Lin Y.H., Eynard A.R., Valentich M.A., Fernandez-Zapico M.E., Pasqualini M.E. (2011). Basic aspects of tumor cell fatty acid-regulated signaling and transcription factors. Cancer Metastasis Rev..

[150] Horne B.D., Muhlestein J.B., Anderson J.L. (2015). Health effects of intermittent fasting: Hormesis or harm? A systematic review. Am. J. Clin. Nutr..

[151] Piercy K.L., Troiano R.P., Ballard R.M., Carlson S.A., Fulton J.E., Galuska D.A., George S.M., Olson R.D. (2018). The Physical Activity Guidelines for Americans. JAMA.

[152] Ligibel J. Pilot Study of the Impact of a Combined Intermittent Fasting and Exercise Intervention on Metabolic Markers in Patients with Advanced, Hormone Receptor Positive Breast Cancer. https://clinicaltrials.gov/ct2/show/NCT04708860.

[153] Varady K. Alternate Day Fasting Combined with Exercise for the Treatment of Non-Alcoholic Fatty Liver Disease (NAFLD). https://clinicaltrials.gov/ct2/show/NCT04004403.

[154] Ryan A. Promotion of Successful Weight Management in Overweight and Obese Veterans. https://clinicaltrials.gov/ct2/show/NCT04131647.

[155] Risa Ø. Before the Beginning: Preconception Lifestyle Interventions to Improve Future Metabolic Health. https://clinicaltrials.gov/ct2/show/NCT04585581.

[156] Sungkarat S. A Randomized Controlled Trial Investigating the Effects of Combined Physical-Cognitive Exercise and Dietary Intervention on Cognitive Performance and Changes in Blood Biomarkers of Postmenopausal Obese Women. https://clinicaltrials.gov/ct2/show/NCT04768725.

[157] Zhu Y. Effects of Diet and Exercise Interventions on Cardiometabolic Risk Markers, Executive Function, and Intestinal Flora in Undergraduate Students: A Randomized Controlled Trial. https://clinicaltrials.gov/ct2/show/NCT04834687.

[158] Gabel K. Time Restricted Eating with Physical Activity for Weight Management. https://clinicaltrials.gov/ct2/show/NCT04978376.

